# The Medical Law

**Published:** 1896-03

**Authors:** 


### The Medical Law.

A law regulating the practice of medicine in Ohio has just been enacted by our State Legislature. Two years ago a bill embracing substantially the same provisions as the present law was presented to the Legislature and met with ignominious defeat in both houses. It met with such treatment at the hands of the

Legislature as to arouse the indignant protest of almost every honorable medical professional man in the State, and this resulted in a great degree, by the efforts of physicians, in such a change in the personnel of the Legislature as to attain the end recently achieved.

It was enacted by well-nigh the unanimous vote of both houses, which is a severe rebuke to that body of two years ago, at whose hands the same bill received such shameful treatment.

This law is a most excellent one, in all its provisions, and will have a good influence in freeing the State from that overwhelming mass of quackery and charlatanism that have so long run a rampant course throughout the State.

It will be a matter of interest to the dentists of the State to note the means that shall be employed for the efficient execution of this law. We may thereby learn a lesson. The results of this medical law will be watched with great interest, not only by the medical profession, but by the specialist of that profession, and by every philanthropist and well-wisher of his kind throughout the State. It will only call forth regret and sorrow from those who have been bringing discredit upon a noble profession, robbing the people of their money, and tampering in a most reprehensible way with the health and welfare of the public and bringing a noble profession into disgrace.

